# Comparative Transcriptome Analysis of Shiga Toxin-Producing *Escherichia coli* O157:H7 on Bovine Rectoanal Junction Cells and Human Colonic Epithelial Cells during Initial Adherence

**DOI:** 10.3390/microorganisms11102562

**Published:** 2023-10-15

**Authors:** Lekshmi K. Edison, Indira T. Kudva, Subhashinie Kariyawasam

**Affiliations:** 1 Department of Comparative, Diagnostic, and Population Medicine, College of Veterinary Medicine, University of Florida, Gainesville, FL 32610, USA; edison.le@ufl.edu; 2 Food Safety and Enteric Pathogens Research Unit, National Animal Disease Center, Agricultural Research Service, U.S. Department of Agriculture, Ames, IA 50010, USA; indira.kudva@usda.gov

**Keywords:** adherence, biofilm formation, colonic epithelium, rectoanal junction, Shiga toxin-producing *Escherichia coli*, transcriptomics, virulence genes

## Abstract

Shiga toxin-producing *Escherichia coli* (STEC) are notorious foodborne pathogens, capable of causing severe diarrhea and life-threatening complications in humans. Cattle, acting as both primary reservoirs and asymptomatic carriers of STEC, predominantly harbor the pathogen in their rectoanal junction (RAJ), facilitating its transmission to humans through contaminated food sources. Despite the central role of cattle in STEC transmission, the molecular mechanisms governing STEC’s adaptation in the RAJ of the asymptomatic reservoir host and its subsequent infection of human colonic epithelial cells, resulting in diarrhea, remain largely unexplored. This study aims to uncover these complicated dynamics by focusing on the STEC O157:H7 serotype within two distinct host environments, bovine RAJ cells and human colonic epithelial cells, during initial colonization. We employed comparative transcriptomics analysis to investigate differential gene expression profiles of STEC O157:H7 during interactions with these cell types. STEC O157:H7 was cultured either with bovine RAJ cells or the human colonic epithelial cell line CCD CoN 841 to simulate STEC-epithelial cell interactions within these two host species. High-throughput RNA sequencing revealed 829 and 1939 bacterial genes expressed in RAJ and CCD CoN 841, respectively. After gene filtering, 221 *E. coli* O157:H7 genes were upregulated during initial adherence to CCD CoN cells and 436 with RAJ cells. Furthermore, 22 genes were uniquely expressed with human cells and 155 genes with bovine cells. Our findings revealed distinct expression patterns of STEC O157:H7 genes involved in virulence, including adherence, metal iron homeostasis, and stress response during its initial adherence (i.e., six hours post-infection) to bovine RAJ cells, as opposed to human colonic epithelial cells. Additionally, the comparative analysis highlighted the potential role of some genes in host adaptation and tissue-specific pathogenicity. These findings shed new light on the potential mechanisms of STEC O157:H7 contributing to colonize the intestinal epithelium during the first six hours of infection, leading to survival and persistence in the bovine reservoir and causing disease in humans.

## 1. Introduction

Shiga toxin-producing *Escherichia coli* (STEC) are highly virulent foodborne pathogens that pose a significant threat to public health worldwide and are responsible for numerous outbreaks of severe gastrointestinal infections [1,2]. Infection with this pathogen can lead to a spectrum of symptoms, ranging from mild diarrhea to life-threatening complications, such as hemolytic uremic syndrome (HUS) and hemorrhagic colitis (HC) [2,3]. The major route of transmission to humans is through the consumption of contaminated food, particularly undercooked beef and dairy products [4,5]. While STEC can asymptomatically colonize the gastrointestinal tracts of healthy cattle and other ruminants, the mechanisms by which it adapts to its ruminant reservoir and subsequently causes infections in humans are still not fully understood. In the United States, disease outbreaks often result from the consumption of contaminated bovine food products and fresh produce which have been tainted with the bacterium due to exposure to bovine waste [6].

The bovine rectoanal junction (RAJ) has emerged as a critical anatomical site for STEC colonization in cattle [7]. The RAJ is a transition zone between the rectum and the anal canal, composed of columnar and squamous cells, and characterized by a unique microenvironment that favors bacterial colonization and persistence [8,9]. Consequently, the RAJ serves as a major reservoir for STEC, and the contamination of food products can occur through fecal shedding from infected cattle during the slaughtering process [10,11]. The adaptation of STEC to the bovine RAJ microenvironment likely involves the differential regulation of specific genes and virulence factors that facilitate bacterial adherence, survival, and evasion of the host immune system. Conversely, during human infections, STEC encounters a vastly different environment in the colonic epithelial cells of the human gut. The pathogen must overcome numerous host defenses, including the mucus layer, antimicrobial peptides, and the gut microbiota, to colonize and cause disease [12,13]. During this process, STEC is believed to differentially regulate gene expression to promote its survival, colonization, and pathogenicity in the human colonic epithelium.

Understanding the molecular mechanisms behind STEC disease pathogenesis is crucial for the development of targeted therapies and preventive measures to combat this dangerous foodborne pathogen. The adherence mechanisms employed by STEC are pivotal in their ability to initiate and establish infections [14]. It is well known that the intimin protein, encoded by *eae*, is a crucial virulence factor that mediates intimate attachment of STEC by binding to a receptor called Tir on the host cell [15]. This interaction leads to the formation of attaching and effacing (A/E) lesions, characterized by localized destruction of host cell microvilli and intimate bacterial adhesion. However, the adhesins involved in initial adherence to STEC, leading to subsequent intimate adherence, are largely unknown. This is despite the fact that several fimbrial and non-fimbrial adhesins of STEC have been implicated in STEC initial adherence to host cells [16,17,18]. Previous research also suggests the carbon salvation protein Slp (carbon starvation-inducible lipoprotein) might be involved in the initial adherence of STEC to human colonic epithelial cells [19]. These adherence mechanisms may not only facilitate bacterial colonization but also enable the pathogens to evade host defenses and deliver their virulence factors, ultimately contributing to the severity of STEC infections.

Transcriptomics, which enables the genome-wide analysis of gene expression patterns, has become an indispensable tool in understanding the intricate interplay between pathogens and their hosts [20]. Comparative transcriptomics analysis allows for the identification of differentially expressed genes and pathways in response to specific environmental conditions, thereby offering valuable insights into the molecular basis of host adaptation and pathogenesis [21]. Despite the potential of transcriptomics to shed light on the mechanisms underlying STEC’s adaptation to different host environments, only limited studies have focused on the comparative transcriptomic analysis of this pathogen grown on bovine RAJ cells and human colonic epithelial cells. In this study, we aimed to address this knowledge gap by conducting a comprehensive comparative transcriptomics analysis of STEC O157:H7, the most studied serotype of STEC, cultivated on both bovine RAJ and human colonic epithelial cells. High-throughput RNA sequencing was utilized to profile the global gene expression patterns of STEC O157:H7 under these two distinct host environments. Through this approach, we intended to identify the key genes and regulatory pathways that are differentially expressed in response to the bovine and human host environments, holding significant promise for enhancing our understanding of STEC pathogenesis and host adaptation. By unraveling the genetic responses of STEC O157:H7 during initial adherence to bovine RAJ and human colonic epithelial cells, our goal was to uncover the key molecular mechanisms that underlie its ability to colonize and cause disease in these disparate host environments. Ultimately, this research will pave the way for targeted interventions and preventive strategies for this important foodborne pathogen.

## 2. Materials and Methods

### 2.1. Bacterial Strains 

*E. coli* O157:H7 wild-type (O157 WT) EDL 932 (American Type Culture Collection/ATCC, Manassas, VA, USA), which is positive for *stx1*, *stx2*, and *eae* was used in this study. Bacteria were grown at 37 °C in Luria Bertani (LB) (BD, Difco, Franklin Lakes, NJ, USA) broth or LB agar plates without antibiotics.

### 2.2. Cell Culture and Media

The CCD CoN 841 human colonic epithelial cells (ATCC CRL-1790), purchased from ATCC (Manassas, VA, USA), were grown in Eagle’s Minimum Essential Medium (EMEM; ATCC, Manassas, VA, USA) containing 10% fetal bovine serum (FBS; Atlanta Biologicals, Flowery Branch, GA, USA) in the presence of 5% CO_2_ at 37 °C. RAJ cells, including both squamous and columnar cells, were isolated from healthy cattle using a previously standardized protocol [22,23] and evaluated similarly at 37 °C in the presence of 5% CO_2_ in Dulbecco’s Modified Eagle’s Medium-High Glucose (DMEM-HG; ATCC, Manassas, VA, USA) containing 10% FBS. 

### 2.3. Bacterial Infection and Adherence

The CCD CoN 841 cells and RAJ cells were tested in tissue culture-treated six-well plates (Corning Life Sciences, Tewksbury, MA, USA) at a seeding density of 10^6^ cells per well. The cells were rinsed with sterile phosphate-buffered saline (PBS), and fresh cell culture media was added 2 to 4 h before infecting the cells with bacteria. To infect the cells, *E. coli* O157:H7 from overnight culture was washed with PBS, resuspended in respective cell growth media, and added at a multiplicity of infection (MOI) of 20 per well. Following incubation for six hours, the cells were gently washed three times with sterile PBS to remove unadhered bacteria. Cells with adhered bacteria were collected using a sterile cell scraper, and wells were rinsed with 1 mL sterile PBS to ensure collection of all components. Collected samples were centrifuged at 12,000× *g* for 10 min, and pellets were stored in RNA*later* RNA stabilization solution (Invitrogen, Waltham, MA, USA). 

### 2.4. Isolation of Bacterial RNA from Infected Cells and mRNA Enrichment

Total RNA was extracted using the RiboPure RNA Purification Kit (Invitrogen, Waltham, MA, USA). Bacterial RNA was separated from total RNA by depleting the mammalian RNA using the MICROBEnrich Kit (Invitrogen, Waltham, MA, USA). Host-depleted samples were further processed for rRNA depletion to enrich for bacterial mRNA using the MICROBExpress Bacterial mRNA Enrichment Kit (Invitrogen, Waltham, MA, USA). All steps were carried out according to the manufacturer’s instructions. Quality and concentration of RNA samples in each step were measured using Qubit™ 4.0 Fluorometer (Thermo Fisher Scientific, Wilmington, DE, USA).

### 2.5. Illumina Library Preparation and RNA Sequencing

Library preparation and RNA sequencing were performed on an Illumina platform through Genewiz (South Plainfield, NJ, USA). Briefly, the library preparation was performed using a NEBNext Ultra RNA Library Prep Kit (Illumina Inc, San Diego, CA, USA) as per the manufacturer’s recommendations. Enriched mRNAs were fragmented, first-strand and second-strand cDNA were synthesized, and fragments were end-repaired, adenylated at 3′ ends, and ligated with universal adapters. The mRNA libraries were multiplexed and clustered on one lane of a flow cell for sequencing on a HiSeq 2500 (Illumina) platform with 2 × 100 paired-end (PE) reads. *E. coli* O157 incubated for six hours in EMEM and DMEM-HG media only without cells were used as controls.

### 2.6. Sequence Analysis and Detection of Differential Gene Expression

Quality checking, base trimming, read mapping, normalization, and differential gene expression analysis were performed using the CLC Genomics Workbench version 22.0.3 (QIAGEN Inc., Redwood City, CA, USA). Raw read sequences were imported into the CLC Genomics Workbench and aligned to *E. coli* O157:H7 strain EDL933 (NCBI RefSeq assembly: GCF_000006665.1) chromosome (NC_002655.2) as well as the associated plasmid pO157 (NC_007414.1). Gene expression was calculated using the RPKM (reads per kilobase of exon model per million mapped reads), and applying the equation RPKM = number of gene reads/(mapped reads (millions) × gene length (kb)). False discovery rate (FDR) [24] was applied to identify statistically significant alterations in gene expression. To identify differential expression (DE) in two groups (treated vs. control), TMM Normalization (Trimmed Mean of M values) described for Whole-Transcriptome RNA-seq Technology was applied. Samples were submitted in triplicate, and the results were averaged to obtain fold changes (FC). Three gene filtering criteria were applied to differentially expressed data: FDR ≤ 0.05, FC ≥ 2.0, and *p*-value ≤ 0.01. Gene enrichment analysis was performed using ShinyGO 0.77 and iDEP 1.1 [25]. Heat maps were created using iDEP 1.1, applying the Euclidean distance with average linkage clustering. 

### 2.7. Statistical Analysis, Software, and Data Preparation

Statistical analysis of differential expressions was performed with the CLC Genomics Workbench using General Linear Model with negative binominal distribution. *p*-values of <0.05 were considered statistically significant. The graphs were plotted using GraphPad Prism version 10.0.1 (Graphpad Software Inc., San Diego, CA, USA). All quantitative analysis was performed in triplicate in independent experiments. 

### 2.8. Data Availability

The transcriptomic profile data (both raw and processed) described in this study were deposited in the Gene Expression Omnibus (GEO) database in NCBI, under the accession number GSE240423. 

## 3. Results

### 3.1. Overall Transcriptomics Profiles of E. coli O157:H7 Cultured with CCD CoN 841 and RAJ Cells during Initial Adherence 

**Figure 1:**
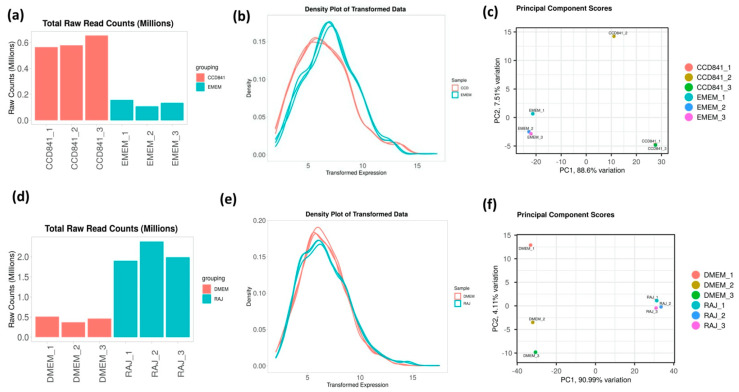
Diagnostic plots depicting the sequencing depth bias in each treatment. (**a**,**d**) Total raw read counts per library. (**b**,**e**) Density plot of regularized log-transformed total read counts. (**c**,**f**) Differentiation between treated and control samples is readily apparent in the principal component analysis (PCA), which accounts for 88.6 and 91.26% of the observed variation in CDD CoN 841 and RAJ-infected *E. coli* O157:H7, respectively, when compared to untreated samples. Total read counts are significantly different among sample groups (*p* = 1.16 × 10^−4^) based on ANOVA.

To determine the differential gene expression of *E. coli* O157:H7 during initial adherence to the intestinal epithelia of bovine and human hosts, CCD CoN 841 and RAJ cells were infected with *E. coli* O157:H7 for six hours. Total RNA with RIN (RNA Integrity Number) values between 8.7 and 9.5 were enriched for bacterial RNA and depleted of rRNA. The resultant bacterial mRNA was subjected to deep sequencing at 100 million reads coverage. The obtained transcript reads successfully mapped to the *E. coli* O157:H7 strain EDL933 genome. The percentage coverages of map reads were 87.7 for CCD CoN 841/O157:H7 and 88.18 for RAJ/O157:H7. For the respective controls, the percent coverages were 80.53 and 83.18 for *E. coli* O157:H7 grown in EMEM and DMEM-HG, respectively. Diagnostic plots for read count data were generated for analyzing the variations in the mapped library sizes (Figure 1). 

### 3.2. Overall Transcriptomics Differences of E. coli O157:H7 Cultured with CCD CoN 841 and RAJ Cells during Initial Adherence

**Figure 2:**
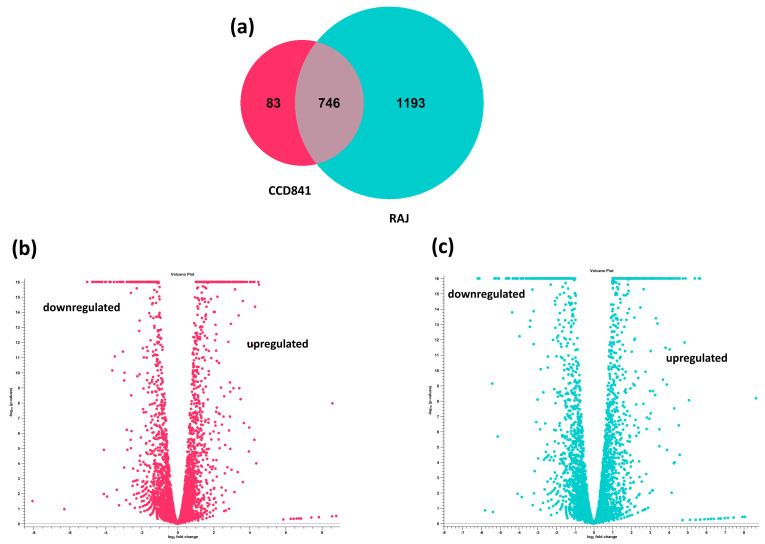
Differential transcriptome profiles of *E. coli* O157:H7 in CCD CoN 841 and RAJ cells (total transcriptomic data without filtration). (**a**) Venn diagram showing unique and differentially expressed genes. Volcano plots showing differentially expressed *E. coli* O157:H7 genes in CCD CoN 841 cells (**b**) and RAJ cells (**c**). In volcano plots, the relationship between *p*-values and the magnitude of the difference in expression values was noted. *X*-axis indicates log_2_ fold change threshold, and *Y*-axis depicts −log10 (*p*-values).

The transcriptome of *E. coli* O157:H7 was completely different in the human colon cell line compared to the bovine RAJ cells during initial adherence at six hours. Differential expression analysis was performed on the CLC Genomics Expression Browser tool, and the results are provided in Supplementary File S1 (*E. coli* O157:H7 in CCD CoN 841 vs. EMEM media control) and Supplementary File S2 (*E. coli* O157:H7 in RAJ vs. DMEM-HG media control). The total number of expressed genes of *E. coli* O157:H7 in CCD CoN 841 cells and RAJ cells were 829 and 1939, respectively. A total of 83 *E. coli* O157:H7 genes were uniquely expressed in CCD CoN 841 cells, whereas 1193 genes were uniquely expressed in RAJ cells. Another 746 genes were expressed commonly in both cell types (Figure 2). A total of 417 *E. coli* O157:H7 genes were upregulated and 412 were downregulated in CCD CoN 841 cells, whereas 1024 genes were upregulated and 915 were downregulated in RAJ-adhered *E. coli* O157:H7. Differential changes in transcriptomes are represented by volcano plots (Figure 2). Heatmaps of expression profiles of all differentially expressed genes are depicted in Supplementary Figure S1. To investigate the comparative analysis of transcriptomic shifts, the differentially expressed genes (DEGs) were filtered using criteria, such as two-fold change, FDR ≤ 0.05, and *p*-value ≤ 0.01.

### 3.3. The Enriched Biological Pathways during the Initial Adherence of E. coli O157:H7 in CCD CoN 841 and RAJ Cells 

**Figure 3:**
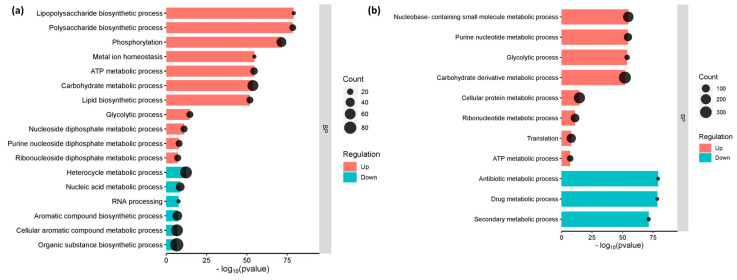
Enriched functional categories of differentially expressed genes. (**a**) *E. coli* O157:H7 attached to CCD CoN 841 cells. (**b**) *E. coli* O157:H7 attached to RAJ cells. The *x*-axis is on log_10_ (*p*-value) scale. BP denotes ‘biological process.’.

The gene enrichment analysis, which categorized the genes into their respective metabolic function, has shed light on the cellular and molecular processes that underlie interactions between *E. coli* O157:H7 and host cells. Genes involved in lipopolysaccharide biosynthesis, polysaccharide biosynthesis, lipid biosynthesis, and metal ion homeostasis were considerably elevated in *E. coli* O157:H7 adhered to CCD CoN 841 cells (*p*-value 0.01 and FDR 0.05). However, processes involved in the production of aromatic and organic compounds, as well as heterocycles, were suppressed, whereas *E. coli* O157:H7 attached to RAJ cells showed considerable downregulation in metabolic pathways involving antibiotics and secondary metabolites. Both treatments showed similar enrichment for all other cellular metabolic pathways (Figure 3). 

### 3.4. Functional Annotation of E. coli O157:H7 Genes Differentially Expressed in Two Different Host Cell Types 

**Figure 4:**
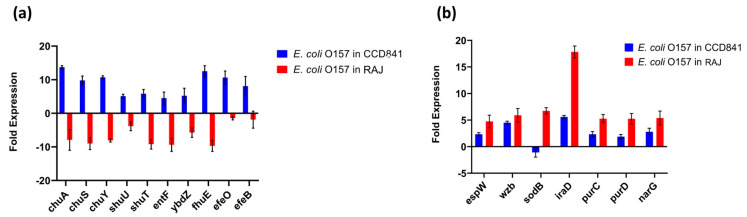
Expression patterns of differentially expressed genes. (**a**) Upregulated virulence genes in *E. coli* O157:H7 adhered to CCD CoN 841. (**b**) Highly expressed virulence genes in *E. coli* O157:H7 during RAJ adhesion. Virulence genes were identified in a search with the virulence factor database (VFDB) (http://www.mgc.ac.cn/VFs/main.htm, accessed on 14 July 2023). Gene expression data represent the mean of three replicates ± standard deviation. Please note that some house-keeping genes were also included in the VFDB.

The top 20 genes upregulated in each treatment group (i.e., two host cell types) with their fold expression, subcellular location, and functional category are listed in Table 1. Specifically, 10 *E. coli* O157:H7 genes implicated in iron acquisition, utilization, and survival were upregulated during initial adherence to human colonic epithelial cell line CCD CoN 841. However, all these genes were downregulated during adherence to bovine RAJ cells (Figure 4a). These included three genes involved in the iron transport locus *chu* (*chuA*, *chuS*, and *chuY*), two genes in the *shu* heme utilization locus (*shuU* and *shuT*), the enterobactin synthase multienzyme complex component genes *entF* and *ybdZ* involved in the enterobactin biosynthetic process, the high-affinity ferric uptake transmembrane transporter *fhuE*, and the iron uptake and assimilation genes *efeB* and *efeO*. 

This study unveiled the presence of seven virulence genes, namely *espW* (Type III secretion system effector), *wzb* (capsular polysaccharide biosynthesis and assembly), *iraD* (regulating flagellar synthesis), *purC* and *purD* (purine biosynthesis), and *narG* (nitrate reductase) expressed in *E. coli* O157:H7 on both cell types. The expression was much higher in bovine cells than in human cells. In contrast, the gene encoding superoxide dismutase, *sodB*, was upregulated in the bovine context and downregulated in human cells (Figure 4b).

**Figure 5:**
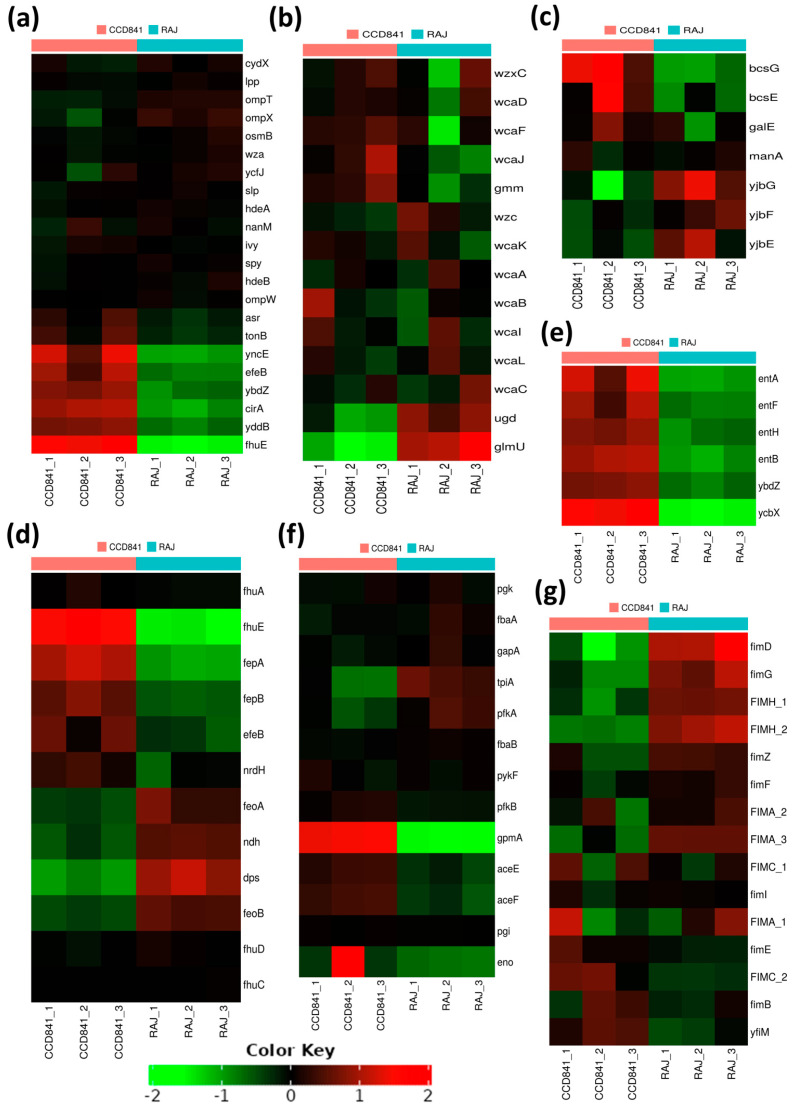
Heat maps of transcriptional responses of *E. coli* O157:H7 during the initial adherence of human CCD CoN 841 cells and bovine RAJ cells based on functional analysis. (**a**) Outer membrane proteins. (**b**) Lipopolysaccharide metabolic processes. (**c**) Polysaccharide biosynthesis. (**d**) Cellular homeostasis. (**e**) Antibiotic metabolic processes and secondary metabolism. (**f**) Glycolytic pathway. (**g**) Cell adhesion or fimbriae/pilus assembly and regulation. Transformed FPKM (Fragments Per Kilobase of Transcript per Million) numbers (normalized read counts) are represented by colors, with green indicating lower expression and red indicating higher expression.

We also noted 22 genes encoding outer membrane proteins (OMPs), out of which 15 consistently expressed at equal levels in *E. coli* O157:H7 grown with either bovine or human cells. In contrast, the remaining seven OMP-encoding genes, *fhuE*, *yddB*, *cirA*, *ybdZ*, *eleB*, *yncE*, and *asr*, displayed substantial and notable upregulation, specifically in *E. coli* O157:H7 grown with human CCD CoN 841 cells (Figure 5a). Biofilm formation and other processes related to lipopolysaccharide metabolism were upregulated in both treatment groups. These included (i) a group of genes associated with the biosynthesis of colanic acid and a component of the extracellular matrix (*wcaA*, *wcaB*, *wcaC*, *wcaD*, *wcaI*, *wcaJ*, *wcaF*, *wcaL*, *wcaK*, *ugd*, and *wzc*); (ii) *ugd*, *wcaC*, and *glmU*, involved in the biosynthesis of UDP-N-acetylglucosamine (UDP-GlcNAc); (iii) *lipA* (biotin biosynthesis) and *fabI* (fatty acid beta-oxidation) (Figure 5b); and (iv) *yjbG*, *yjbE*, and *yjbF*, involved in extracellular polysaccharide production during biofilm formation (Figure 5c). 

Most genes related to cellular homeostasis exhibited upregulation in CCD CoN 841-adherent *E. coli* O157:H7, signifying a concerted effort to maintain equilibrium in the human host. For example, numerous genes associated with iron metabolism and homeostasis, including those encoding the iron transporter proteins (*fhuA*, *fhuD*, *fhuC*, *fhuD*, and *fhuE*), were upregulated as compared to *E. coli* O157:H7 grown with RAJ cells. Similarly, the genes participating in cellular response to DNA protection (*fepB*), colicin transport (*fepA*), cellular oxidant detoxification (*efeB*), and cell redox homeostasis (*nrdH*) were also upregulated (Figure 5d). Interestingly, we found a deliberate upregulation of genes involved in antibiotic resistance and secondary metabolic processes during *E. coli* O157:H7 adherence to human CCD841 cells. Specifically, genes linked to the enterobactin transporter and enterobactin synthase components (*entA*, *entB*, *entF*, *entH*, *ybdZ*, and *ycbX*) were activated (Figure 5e). During interaction with the bovine RAJ cells, *E. coli* O157:H7 genes associated with the glycolytic pathway (Figure 5f) and fimbriae/pilus assembly and regulation, such as *fimD*, *fimG*, *fimA_1*, *fimA_2*, *fimA_3*, *fimH_1*, *fimZ*, *fimC_1*, *fimF*, and *fimI* (Figure 5g), were upregulated in comparison to *E. coli* O157:H7 adhered to CCD CoN 841. Likewise, 15 *eut* genes (*eutS*, *eutE*, *eutQ*, *eutK*, *eutC*, *eutM*, *eutH*, *eutF*, *eutG*, *eutC*, *eutB*, *eutK*, *eutL*, *eutT*, and *eutH*), which play pivotal roles in ethanolamine catabolism, demonstrated significantly higher upregulation during initial attachment to bovine RAJ cells compared to the CCD CoN 841 cells. An additional four *eut* genes—namely *eutA*, *eutE*, *eutI*, and *eutP*—were expressed solely in association with bovine RAJ cells.

Our comprehensive analysis, after applying the gene filtering criteria, revealed that a total of 221 and 436 *E. coli* O157:H7 genes were upregulated during initial adherence with CCD CoN and RAJ cells, respectively. In addition to these commonly upregulated genes, a set of 22 genes exhibited unique expression with human cells, while 155 genes displayed unique expression with bovine cells. A detailed list of noteworthy upregulated and uniquely expressed genes in *E. coli* O157:H7, during the initial adherence to CCD CoN 841 and RAJ cells, is included in Supplementary Table S1.

## 4. Discussion

The O157 and non-O157 STEC are prominent foodborne pathogens with global impact, known for inducing severe illnesses in human populations across the world [26,27,28]. STEC has a significant reservoir in healthy cattle, which serve as asymptomatic carriers. Initial attachment to intestinal cells is of pivotal importance, as it marks a crucial early stage in the development of pathogenicity in humans or asymptomatic colonization of cattle [29,30]. Our research aimed to enhance the understanding of transcriptional gene regulation occurring during the initial attachment of STEC O157:H7 to both human and bovine intestinal cells. The goal was to shed new light on the current knowledge of STEC asymptomatic colonization in the bovine host versus pathogenesis in the human host. 

In the context of *E. coli* O157:H7 adherence to CCD CoN 841 cells, the enrichment of the lipopolysaccharide, polysaccharide, and lipid biosynthesis genes highlighted that *E. coli* O157:H7 might strategically manipulate its outer membrane composition to enhance adhesion and potentially establish a stable niche within the host environment within the first six hours of infection [31,32,33]. Furthermore, the enrichment of genes associated with metal ion homeostasis indicates the bacterium’s adaptation to the host cell environment, possibly to ensure its survival and effective colonization [34,35]. The upregulated genes were more noticeable during *E. coli* adherence to CCD CoN 841 cells. However, the downregulation of pathways related to antibiotic, drug, and secondary metabolism were particularly intriguing during their adherence to RAJ cells. This suggests that distinct mechanisms are employed by *E. coli* O157:H7 during adaptation to the reservoir host and the human host. The reduced emphasis on secondary metabolic pathways might reflect the bacterium’s shift in resource allocation toward more immediate requirements, such as adherence and colonization of RAJ to establish a long-term carrier state in the bovine host [36,37].

The findings from the differential expression analysis also provide valuable insights into the dynamic response of STEC O157:H7 during infection, illuminating its strategies for iron acquisition, utilization, and overall survival within distinct host environments. Notably, the observed differential regulation of 10 genes associated with iron-related processes presents a compelling glimpse into the bacterium’s adaptive mechanisms when interacting with different host cell types. In the context of attachment to human colonic epithelial cell line CCD CoN 841, the upregulation of these genes highlights the bacterium’s heightened demand for iron. Iron is an essential nutrient for bacterial growth, and pathogens often employ sophisticated tactics to secure this critical resource from their host [38]. The substantial upregulation of genes involved in iron transport, heme utilization, enterobactin biosynthesis, and high-affinity ferric uptake underscores *E. coli* O157:H7’s concerted efforts to scavenge and efficiently utilize iron from the human colonic environment. This strategic response could potentially contribute to the bacterium’s ability to establish a foothold within the human host and establish conditions conducive to pathogenicity. The five virulence genes *chuA*, *chuS*, *chuY*, *shuU*, and *shuT* are particularly interesting because of the complex interplay between their products in relation to iron transport and heme utilization. The *chuA* gene, which encodes an outer membrane hemoglobin receptor protein crucial for heme uptake, stands out as a pivotal player in the bacterium’s quest for iron. Its differential expression could play a key role in the overall iron acquisition strategy of *E. coli* O157:H7, enabling it to efficiently harvest heme-bound iron from the host environment [39]. This heightened activity of *chuA* within the human colon might contribute to the bacterium’s virulence and ability to cause disease in humans. However, a converse pattern emerged in the bovine RAJ cell infection scenario, where the same set of genes displayed downregulation. This distinct regulatory behavior suggests a tailored approach of *E. coli* O157:H7 when confronted with the bovine host environment. The observed downregulation of these iron-related genes in bovine RAJ-infected *E. coli* O157:H7 implies a different iron usage landscape during initial hours in the bovine host, establishing its mutual existence rather than damaging the host, as is the case within the human host. For example, in a chicken infection model, iron acquisition systems, such as salmochelin and aerobactin, played a more significant role in the virulence of ExPEC than heme utilization [40]. This might reflect the bacterium’s adaptability to varying host contexts.

Infection of human CCD CoN 841 cells by *E. coli* O157:H7 prompted the upregulation of numerous genes associated with cellular homeostasis, particularly those linked to metal iron homeostasis. This included the significant elevation of iron transporter genes, along with genes involved in DNA protection, colicin transport, cellular oxidant detoxification, and cell redox homeostasis, suggesting a coordinated response to host cell interactions. The iron-related genetic response could reflect the bacterium’s adaptation to the host’s iron availability, a critical factor for its survival and growth [41,42]. This particularly intriguing observation emerged as we investigated the behavior of *E. coli* O157:H7 during its adhesion to CCD CoN 841 cells.

The investigation revealed a distinctive set of *E. coli* O157:H7 virulence genes exhibiting upregulation within the bovine RAJ environment. This observation might shed valuable insights into the pathogen’s adaptation and response, depending on the specific host environment. An area that needs further exploration is the upregulation of *espW*, which encodes a vital component of the Type III secretion system (T3SS). The *espW* is predominantly located within the locus of enterocyte effacement (LEE) pathogenicity island, which allows for intimate attachment of typical STEC, like the *E. coli* strain we used in this study, to the intestinal epithelium, causing characteristic attaching and effacing lesions during infection [43]. Previously, Kudva et al. [44] demonstrated that LEE-encoded proteins do not have a role in STEC adherence to squamous cells at the RAJ, although they are needed for the effective adherence to the columnar cells of the RAJ. The EspW effector is responsible for triggering changes in the arrangement of actin filaments within human host cells [43]. However, the role of EspW in colonizing *E. coli* O157:H7 in bovine RAJ cells has yet to be determined. Another gene overly expressed in the context of RAJ cells only was *wzb,* which is involved in the synthesis and assembly of capsular polysaccharides. Capsular polysaccharides play an important role in evading host immune responses and enhancing the pathogen’s ability to colonize and persist within the host [45]. Therefore, they might aid *E. coli* O157:H7 in establishing an overt infection in the bovine host. 

The outer membrane proteins (OMPs) facilitate various processes, such as biofilm formation, infection of eukaryotic cells, antibiotic resistance, and modulation of immune responses [46]. This illustrates that OMPs are essential players in the bacterium’s adaptation to its environment and its interactions with the host [47]. 

The observed differential regulation of 15 OMP-encoding genes in bovine and human intestinal epithelium suggests the adaptability and plasticity of *E. coli* O157:H7 in tailoring its molecular strategies to suit the requirements of both hosts during its initial attachment. Additionally, the seven OMP genes (*fhuE*, *yddB*, *cirA*, *ybdZ*, *eleB*, *yncE*, and *asr*) of *E. coli* O157:H7 upregulated in association with human CCD841 CoN 841 only. This implies a specialized role of these gene products in the human host environment, but not in the bovine host. Understanding the role of these OMPs might open new avenues for exploring potential targets for interventions and therapies to mitigate the impact of *E. coli* O157:H7 infections in both animal and human populations. We also observed upregulation of a set of genes (*wcaA*, *wcaB*, *wcaC*, *wcaD*, *wcaI*, *wcaJ*, *wcaF*, *wcaL*, *wcaK*, *ugd*, and *wzc*) associated with colanic acid biosynthesis in *E. coli* O157 adhered to both human and bovine cells. Colanic acid, a component of the extracellular matrix, plays a pivotal role in biofilm development, contributing to the structural integrity and stability of the biofilm matrix [48]. Along with biotin biosynthesis, lipolate synthesis and fatty acid beta-oxidation were also elevated in both infection scenarios. These metabolic pathways may provide the necessary energy and components for the biosynthesis of biofilm matrix components.

When the differentially expressed genes were examined closely, *E. coli* O157:H7, in the RAJ context, exhibited substantial overexpression of three genes involved in extracellular polysaccharide production (*yjbG*, *yjbE*, and *yjbF*) during biofilm formation. This finding underscores the bacterium’s strategic adaptation to the biofilm lifestyle within the specific bovine RAJ environment, perhaps facilitating its persistence as a colonizer. Extracellular polysaccharides are integral components of the biofilm matrix, contributing to cell–cell adhesion and overall biofilm structure [49]. Genes involved in the biosynthesis of UDP-GlcNAc, a fundamental building block in lipopolysaccharide, also exhibited significant upregulation in *E. coli* O157:H7 attached to bovine RAJ cells. This observation suggests an enhanced metabolic activity related to lipopolysaccharide production, potentially contributing to the modification of the bacterial cell surface and interactions with the environment. Upregulation of genes encoding lipid metabolism and extracellular polysaccharide production provides a comprehensive view of the genetic responses underlying biofilm formation in *E. coli* O157:H7 within both treatment groups. These findings highlight the complex coordination of various metabolic pathways and genetic factors that contribute to establishing and maintaining biofilm structures [50,51]. The insights gained from this study pave the way for a deeper understanding of biofilm-related processes and their potential implications for *E. coli* O157:H7 pathogenicity and survival in diverse host environments.

Notably, *E. coli* genes associated with antibiotic and secondary metabolic processes, including the key components of the enterobactin transporter and enterobactin synthase system, exhibited deliberate upregulation during adherence to human colonic epithelial cells. The activation of these genes signifies a strategic genetic response to acquire nutrients from the host as adaptive tactics for survival and persistence within the human host environment during pathogenesis [52,53,54].

Another intriguing observation was the upregulation of genes that encode *E. coli* type-1 fimbriae during the initial attachment of *E. coli* O157:H7 to bovine RAJ cells. Fimbriae and pili are structures on the surface of bacteria that are essential for host adhesion, colonization, and infection [55]. The fimbriae/pilus assembly genes, including *fimD*, *fimG*, and *fimC*, play pivotal roles in the assembly, structural integrity, and initiation of these adhesive structures. Additionally, *fimH*, *fimZ*, *fimF*, and *fimI* are regulatory genes that contribute to the coordination of fimbrial expression and the bacterial response to environmental cues [56,57,58]. The significant upregulation of these genes in bovine-adhered *E. coli* O157:H7 suggests a specialized response and adaptation to the bovine host environment. Bovines are recognized as a major reservoir for *E. coli* O157:H7, and this bacterium’s ability to effectively adhere to the bovine intestinal epithelium is a critical factor for establishing colonization and potential transmission to humans [6]. The observed upregulation likely enhances the bacterium’s capacity to bind to bovine cells, thereby increasing its likelihood of successful colonization. In fact, *E. coli* O157:H7 type-1 fimbrial genes have been reported to play a role in the attachment of superseded *E. coli* O157:H7 to bovine RAJ squamous epithelial cells in a strain-dependent manner [59]. Further studies could elucidate whether this enhanced adhesion contributes to increased colonization in bovines, potentially exploiting the new knowledge to mitigate STEC colonization of the bovine RAJ, thus reducing human foodborne STEC disease.

The heightened expression of ethanolamine catabolic genes (*eut*) in *E. coli* O157:H7 during its initial adherence to bovine intestinal cells, as compared to human colonic epithelial cells, also is noteworthy. It is known that ethanolamine plays a multifaceted role in the virulence of *E. coli* O157:H7 as a nutrient source and signaling molecule, influencing gene expression and contributing to its ability to cause disease and compete with other bacteria in the gut [60,61]. However, the paradox lies in the fact that, while *E. coli* O157:H7 colonizes bovines, it does not cause overt disease in this context [6]. Therefore, our finding of *eut* gene expression in the context of bovine RAJ cells but not human CCD CoN 841 cells is somewhat contrary to previous reports. Future studies must be directed toward elucidating the role of ethanolamine in the initial adherence of STEC to the bovine RAJ, rather than causing disease in the human host. 

In summary, our findings provide insights into the mechanisms employed by *E. coli* O157:H7 in human and bovine hosts following the first six hours of infection. This study provides a foundation for further investigations into the host–pathogen interactions in two different host milieus, leading to disease in one host, whereas colonization and persistence do not lead to disease in another host.

## 5. Conclusions

This study highlights the differences in STEC gene regulation during initial adherence to human CCD CoN 841 cells and bovine RAJ cells, revealing the bacterium’s remarkable adaptability to diverse host microenvironments. Notably, our findings identified the differential regulation of virulence factors, metabolic pathways, and other cellular responses, underscoring versatile strategies used by STEC for colonization, survival, and potential pathogenesis. Particularly significant was the deliberate upregulation of genes associated with iron acquisition, metabolic pathways, and antibiotic resistance during adherence to human colonic epithelial cells. This is contrary to their significant downregulation in bovine RAJ cells, suggesting the bacterium’s different molecular responses to different host cues. These results contribute to a deeper understanding of *E. coli* O157:H7’s behavior within specific host contexts. In addition, the data provide a foundation for future research aimed at deciphering its colonization and pathogenic mechanisms in different host niches and developing targeted interventions to mitigate impact on both animal and human health. We have initiated additional research, based on our findings, to reduce STEC colonization of bovine RAJ and shedding as a preharvest intervention strategy to reduce STEC foodborne outbreaks.

## Figures and Tables

**Table 1:** List of top 20 genes upregulated in *E. coli* O157:H7 during initial adherence to human CCD CoN 841 cells or bovine RAJ cells.

*E. coli* O157:H7 Adhering to CCD CoN 841 Cells				
Gene	Log_2_ FC	Location	Type	Protein Name
*ndhF*	4.224748	membrane	dehydrogenase	Type II NADH:quinone oxidoreductase
*ynfM*	3.996075	membrane	transporter	Inner membrane transport protein YnfM
*yagU*	3.99386	membrane	unknown	Inner membrane protein YagU
*wcaJ*	3.745511	membrane	transferase	UDP-glucose: undecaprenyl-phosphate glucose-1-phosphate transferase
*fhuE*	3.649405	cell outer membrane	Iron transporter	Hydroxamate siderophore receptor FhuE
*yjbF*	3.632319	membrane	unknown	Uncharacterized lipoprotein YjbF
*wza*	3.417282	cell outer membrane	transporter	Putative polysaccharide export protein Wza
*efeO*	3.411274	periplasmic space	transporter	Iron uptake system component EfeO
*fdhF*	3.283415	cytosol	dehydrogenase	Formate dehydrogenase H
*feoB*	3.260966	membrane	transporter	Fe(2+) transporter FeoB
*wcaD*	3.235622	membrane	unknown	Putative colanic acid polymerase
*slp*	3.206504	cell outer membrane	unknown	Outer membrane protein Slp
*wzc*	3.199261	membrane	kinase	Tyrosine-protein kinase Wzc
*adhE*	3.186165	cytosol	acetaldehyde dehydrogenase	Bifunctional aldehyde-alcohol dehydrogenase AdhE
*yohK*	3.087307	membrane	unknown	Inner membrane protein YohK
*pflB*	3.080942	cytoplasm	transferase	Formate acetyltransferase 1
*hycC*	3.043606	membrane	dehydrogenase	Formate hydrogenlyase subunit 3
*efeB*	3.017162	periplasmic space	ferrochelatase	Deferrochelatase
*glnK*	2.907097	membrane	enzyme regulator	Nitrogen regulatory protein GlnK
*amtB*	2.852313	membrane	transporter	Ammonium transporter AmtB
***E. coli* O157:H7 adhering to RAJ cells**				
*yohJ*	4.832419	membrane	unknown	UPF0299 membrane protein YohJ
*fdhF*	4.422977	cytosol	dehydrogenase	Formate dehydrogenase H
*yagU*	4.28355	membrane	unknown	Inner membrane protein YagU
*hycC*	4.249511	membrane	dehydrogenase	Formate hydrogenlyase subunit 3
*yohK*	4.207073	membrane	unknown	Inner membrane protein YohK
*ynfM*	4.193856	membrane	transporter	Inner membrane transport protein YnfM
*pflB*	4.165908	cytoplasm	transferase activity	Formate acetyltransferase 1
*yjbF*	4.156806	membrane	unknown	Uncharacterized lipoprotein YjbF
*hycD*	3.936897	membrane	unknown	Formate hydrogenlyase subunit 4
*wza*	3.806629	outer membrane	transporter	Putative polysaccharide export protein Wza
*ndh*	3.8042	membrane	dehydrogenase	Type II NADH:quinone oxidoreductase
*btsT*	3.674308	membrane	transporter	Pyruvate/proton symporter BtsT
*adiC*	3.625606	membrane	transporter	Arginine/agmatine antiporter
*wcaJ*	3.532739	membrane	transferase	UDP-glucose:undecaprenyl-phosphate glucose-1-phosphate transferase
*hycF*	3.352964	membrane	dehydrogenase	Formate hydrogenlyase subunit 6
*tpiA*	3.309538	cytoplasm	isomerase	Triosephosphate isomerase
*gapA*	3.309281	cytoplasm	dehydrogenase	Glyceraldehyde-3-phosphate dehydrogenase A
*wzc*	3.280554	membrane	kinase	Tyrosine-protein kinase Wzc
*ynfF*	3.279045	periplasmic space	reductase	Probable dimethyl sulfoxide reductase chain YnfF
*ptsG*	3.210761	membrane	transporter	PTS system glucose-specific EIICB component
*slp*	2.011981	cell outer membrane	unknown	Outer membrane protein Slp

## Data Availability

The transcriptomic profiles (both raw and processed data) described in this study were deposited in the Gene Expression Omnibus (GEO) database in NCBI under the accession number GSE240423.

## Supplementary Materials

The following supporting information can be downloaded at: https://www.mdpi.com/article/10.3390/microorganisms11102562/s1, Supplementary File S1: Differential expression analysis of *E. coli* O157:H7 in CCD CoN 841 vs. EMEM media control performed on CLC Genomics expression browser; Supplementary File S2: Differential expression analysis of *E. coli* O157:H7 in RAJ vs. DMEM-HG media control performed on CLC Genomics expression browser; Figure S1: Heatmaps of expression profiles of all differentially expressed genes; Table S1: List of upregulated and uniquely expressed genes in *E. coli* O157:H7 during initial adherence to both CCD CoN 841 and RAJ cells.
