# Supplementary material for: Comparative Transcriptome Analysis of Shiga Toxin-Producing Escherichia coli O157:H7 on Bovine Rectoanal Junction Cells and Human Colonic Epithelial Cells during Initial Adherence
Source: Microorganisms. 2023 Oct 15;11(10):2562. doi: 10.3390/microorganisms11102562 (PMC10609592; doi:10.3390/microorganisms11102562)
Supplement: Supplementary file 1 [file microorganisms-11-02562-s001.zip › Supplementary Table S1 1-IK edited.pdf]

| Upregulated genes of *E. coli* O157:H7 during adherence to CCD CoN 841 cells | | | |
|---|---|---|---|
| **Symbol** | **FC on CCD CoN 841** | **FC on RAJ** | **Biological Function** |
| *aidB* | 2.97 | -2.05 | cellular response to DNA damage stimulus |
| *dctR* | 6.11 | -1.69 | acidic PH |
| *gadA* | 2.10 | -1.37 | carboxylic acid metabolic process |
| *hutW* | 7.97 | -7.80 | Unknown |
| *hutX* | 11.87 | -19.59 | Unknown |
| *iha_1* | 1.97 | -6.37 | Unknown |
| *iha_2* | 1.85 | -7.69 | Unknown |
| *mntH* | 4.25 | -2.74 | cadmium ion transmembrane transport |
| *nrdE* | 4.61 | -4.96 | deoxyribonucleotide biosynthetic process |
| *nrdF* | 5.11 | -10.06 | deoxyribonucleotide biosynthetic process |
| *nrdI* | 5.40 | -5.80 | protein modification process |
| *pqqL* | 5.24 | -2.54 | Proteolysis |
| *shiA* | 4.12 | -1.85 | shikimate transport |
| *sufB* | 2.07 | -2.03 | iron-sulfur cluster assembly |
| *sufC* | 2.40 | -2.28 | iron-sulfur cluster assembly |
| *tdcB* | 7.26 | -1.97 | L-serine catabolic process |
| *wzz(fepE)* | 3.41 | -6.85 | Unknown |
| *yddA* | 5.13 | -2.93 | transmembrane transport |
| *yddB* | 4.42 | -2.46 | iron coordination entity transport |
| *yegP* | 4.50 | -1.94 | double-strand break repair |
| *yjjZ* | 6.23 | -4.09 | Unknown |
| *yncE* | 5.95 | -5.49 | DNA binding proten (outer membrane) |
| **Uniquely expressed genes of *E. coli* O157:H7 during adherence to CCD CoN 841 cells** | | | |
| *adhE* | 9.10 | 0.00 | Glycolysis |
| *cpdB* | 2.03 | 0.00 | cellular response to DNA damage stimulus |
| *fsa_1* | 9.02 | 0.00 | Unknown |
| *gcvT* | 1.80 | 0.00 | glycine catabolic process |
| *glgS* | 3.10 | 0.00 | negative regulation of bacterial-type flagellum-dependent cell motility/negative regulation of biofilm formation |
| *gpmA* | 5.51 | 0.00 | Glycolysis |
| *kch* | 1.88 | 0.00 | ion transport |
| *metE* | 6.89 | 0.00 | cellular amino acid biosynthetic process |
| *minD* | 1.85 | 0.00 | barrier septum assembly |
| *nrdH* | 5.04 | 0.00 | cell redox homeostasis |
| *raiA* | 2.05 | 0.00 | dormancy process |
| *rmf* | 2.04 | 0.00 | dormancy process |
| *tdcA* | 6.84 | 0.00 | L-threonine catabolic process to propionate |
| *uspF* | 3.95 | 0.00 | cell adhesion |
| *ybaT* | 2.42 | 0.00 | response to pH |
| *ybiI* | 4.24 | 0.00 | positive regulation of secondary metabolite biosynthetic process |
| *yciI* | 3.26 | 0.00 | Unknown |
| *yeaG* | 2.47 | 0.00 | cellular response to nitrogen starvation |
| *yeaH* | 2.66 | 0.00 | Unknown |
| *ygeV* | 3.06 | 0.00 | Unknown |
| *yobF* | 1.82 | 0.00 | cellular response to cell envelope stress |
| *ytfT* | 3.94 | 0.00 | carbohydrate transport |
| **Upregulated genes of *E. coli* O157:H7 during adherence to RAJ cells** | | | |
| *eutS* | -1.29 | 8.29 | ethanolamine catabolic process |
| *sodB* | -1.09 | 6.66 | cellular response to chemical stimulus |
| *pta_2* | -1.20 | 5.06 | Unknown |
| *emrK* | -1.00 | 4.91 | drug transmembrane export/ response to antibiotic |

| | | | |
|---|---|---|---|
| *eutE* | -1.99 | 4.04 | Unknown |
| *eutQ* | -1.24 | 3.86 | ethanolamine catabolic process |
| *narK* | -1.19 | 3.82 | nitrate assimilation / transmembrane transport |
| *yfcC* | -1.08 | 3.78 | transmembrane transport |
| *eutM* | -2.32 | 3.53 | ethanolamine catabolic process |
| *ynfH* | -1.42 | 3.05 | anaerobic electron transport chain |
| *napF* | -1.02 | 2.99 | response to oxidative stress |
| *eutG* | -1.53 | 2.97 | alcohol dehydrogenase (NAD) activity |
| *eutC* | -1.20 | 2.92 | ethanolamine catabolic process |
| *cesD* | -1.22 | 2.74 | Unknown |
| *ynjE* | -1.24 | 2.65 | Unknown |
| *eutB* | -1.49 | 2.54 | ethanolamine catabolic process |
| *eutK* | -1.34 | 2.40 | ethanolamine catabolic process |
| *eutL* | -1.36 | 2.40 | ethanolamine catabolic process |
| *fldA* | -1.02 | 2.34 | electron transport chain |
| *eutT* | -2.31 | 2.33 | ethanolamine catabolic process |
| *suhB* | -1.49 | 2.33 | inositol metabolic process/ phosphatidylinositol phosphorylation |
| *cesD2* | -1.22 | 2.31 | Unknown |
| *nrfE* | -1.01 | 2.31 | cytochrome c-heme linkage via heme-L-cysteine |
| *eutH* | -1.13 | 2.27 | ethanolamine catabolic process |
| *fumB_2* | -1.44 | 2.25 | Unknown |
| *nrfG* | -1.21 | 2.14 | cytochrome c-heme linkage via heme-L-cysteine |
| **Uniquely expressed genes of *E. coli* O157:H7 during adherence to RAJ cells** | | | |
| *abrB* | 0.00 | 2.18 | regulation of gene expression |
| *accA* | 0.00 | 1.46 | fatty acid biosynthetic process |
| *accD* | 0.00 | 1.99 | fatty acid biosynthetic process |
| *acrA* | 0.00 | 1.53 | response to toxic substance |
| *adiA* | 0.00 | 3.09 | arginine catabolic process |
| *aegA* | 0.00 | 2.67 | urate catabolic process |
| *allR* | 0.00 | 1.73 | cellular response to DNA damage stimulus |
| *amiD* | 0.00 | 1.61 | cell wall organization/peptidoglycan catabolic process ) |
| *apbE* | 0.00 | 1.53 | protein flavinylation |
| *cpoB* | 0.00 | 1.62 | FtsZ-dependent cytokinesis |
| *cueO* | 0.00 | 1.57 | detoxification of copper ion |
| *cvpA* | 0.00 | 2.16 | toxin biosynthetic process |
| *cyaY* | 0.00 | 3.26 | iron-sulfur cluster assembly |
| *cybB* | 0.00 | 1.49 | removal of superoxide radicals |
| *dinF* | 0.00 | 1.45 | transmembrane transport /xenobiotic transport |
| *dmsB_1* | 0.00 | 6.25 | Unknown |
| *dmsB_2* | 0.00 | 9.29 | Unknown |
| *dtpD* | 0.00 | 1.80 | dipeptide transmembrane transport |
| *ecpR* | 0.00 | 2.37 | regulation of bacterial-type flagellum-dependent cell motility |
| *elaA* | 0.00 | 2.18 | UDP-N-acetylglucosamine biosynthetic process |
| *emrA* | 0.00 | 1.55 | drug transmembrane export/response to toxic substance |
| *emrK* | 0.00 | 4.91 | drug transmembrane export/response to toxic substance |
| *emrR* | 0.00 | 1.60 | Unknown |
| *epmA* | 0.00 | 2.97 | cellular response to acidic pH |
| *eutA* | 0.00 | 2.49 | ethanolamine catabolic process |
| *eutE* | 0.00 | 4.04 | ethanolamine catabolic process |
| *eutJ* | 0.00 | 10.93 | ethanolamine catabolic process |
| *eutP* | 0.00 | 9.11 | ethanolamine catabolic process |
| *fetA* | 0.00 | 2.40 | cellular iron ion homeostasis |
| *fetB* | 0.00 | 2.71 | cellular iron ion homeostasis |

| | | | |
|---|---|---|---|
| *ftnA* | 0.00 | 8.45 | cellular iron ion homeostasis |
| *fur* | 0.00 | 1.53 | negative regulation of siderophore biosynthetic process |
| *glxR* | 0.00 | 3.81 | allantoin assimilation pathway |
| *grlA* | 0.00 | 2.30 | Unknown |
| *grlR* | 0.00 | 3.07 | Unknown |
| *grxA* | 0.00 | 8.87 | cellular response to oxidative stress |
| *grxD* | 0.00 | 2.56 | cellular response to oxidative stress |
| *gsiD* | 0.00 | 1.51 | dipeptide transmembrane transport |
| *gspL* | 0.00 | 1.46 | protein secretion by the type II secretion system |
| *gstA* | 0.00 | 1.61 | response to hydrogen peroxide |
| *hslJ* | 0.00 | 2.33 | response to heat |
| *iraD* | 0.00 | 13.90 | cellular response to oxidative stress |
| *kdpC* | 0.00 | 3.25 | potassium ion transport |
| *lacY* | 0.00 | 2.17 | carbohydrate transmembrane transport |
| *lolB* | 0.00 | 1.75 | lipoprotein localization to outer membrane |
| *lpxA* | 0.00 | 1.87 | lipid A biosynthetic process |
| *lpxC* | 0.00 | 1.57 | lipid A biosynthetic process |
| *lpxD* | 0.00 | 1.47 | lipid A biosynthetic process |
| *marB* | 0.00 | 3.34 | OMP/cellular response to antibiotics |
| *marR* | 0.00 | 1.83 | OMP/cellular response to antibiotics |
| *menB* | 0.00 | 2.65 | menaquinone biosynthetic process |
| *menC* | 0.00 | 2.38 | menaquinone biosynthetic process |
| *menD* | 0.00 | 1.53 | menaquinone biosynthetic process |
| *menE* | 0.00 | 1.64 | menaquinone biosynthetic process |
| *menI* | 0.00 | 1.78 | menaquinone biosynthetic process |
| *mipA* | 0.00 | 2.18 | peptidoglycan biosynthetic process |
| *mlaA* | 0.00 | 1.55 | phospholipid transport |
| *mlaB* | 0.00 | 1.55 | phospholipid transport |
| *mlaC* | 0.00 | 1.53 | phospholipid transport |
| *modE* | 0.00 | 1.76 | molybdate ion transport |
| *mrcB* | 0.00 | 1.54 | cell wall organization /cell wall repair |
| *murA* | 0.00 | 1.47 | UDP-N-acetylgalactosamine biosynthetic process |
| *murI* | 0.00 | 1.90 | cell wall organization |
| *narH_1* | 0.00 | 3.04 | unknown |
| *narI_1* | 0.00 | 4.17 | unknown |
| *narJ* | 0.00 | 3.17 | chaperone-mediated protein complex assembly / nitrate assimilation |
| *narK* | 0.00 | 3.82 | nitrate assimilation |
| *nikB* | 0.00 | 2.40 | dipeptide transmembrane transport |
| *nikC* | 0.00 | 2.08 | dipeptide transmembrane transport |
| *nikD* | 0.00 | 4.06 | dipeptide transmembrane transport |
| *nikE* | 0.00 | 2.00 | nickel cation transport |
| *ompA* | 0.00 | 1.87 | ion transmembrane transport |
| *ompR* | 0.00 | 1.73 | phosphorelay signal transduction system |
| *oppA* | 0.00 | 1.54 | oligopeptide transport |
| *oppC* | 0.00 | 1.49 | oligopeptide transport |
| *oppD* | 0.00 | 1.71 | oligopeptide transport |
| *oppF* | 0.00 | 1.81 | oligopeptide transport |
| *pgaD* | 0.00 | 2.08 | cell adhesion involved in biofilm formation |
| *pgsA* | 0.00 | 1.60 | glycerophospholipid biosynthetic process |
| *potD* | 0.00 | 1.59 | polyamine transport |
| *potE* | 0.00 | 8.92 | L-ornithine transmembrane transport |
| *preA* | 0.00 | 2.00 | bacterial-type flagellum-dependent swarming motility |
| *pta_2* | 0.00 | 5.06 | unknown |

| | | | |
|---|---|---|---|
| *rclA* | 0.00 | 3.35 | response to hypochlorite |
| *rcnB* | 0.00 | 1.66 | cellular response to DNA damage stimulus |
| *rcsB* | 0.00 | 1.49 | cellular stress response to acidic pH |
| *rfaC* | 0.00 | 1.63 | lipopolysaccharide biosynthetic process |
| *rfaD* | 0.00 | 2.14 | lipopolysaccharide biosynthetic process |
| *rfaF* | 0.00 | 1.78 | lipopolysaccharide biosynthetic process |
| *rfaL* | 0.00 | 1.78 | lipopolysaccharide biosynthetic process |
| *rfaY* | 0.00 | 1.50 | lipopolysaccharide biosynthetic process |
| *skp* | 0.00 | 2.10 | Gram-negative-bacterium-type cell outer membrane assembly |
| *slmA* | 0.00 | 1.46 | barrier septum site selection |
| *slyB* | 0.00 | 1.48 | Outer membrane lipoprotein |
| *sodB* | 0.00 | 6.66 | removal of superoxide radicals |
| *sodC_3* | 0.00 | 2.05 | unknown |
| *sorE* | 0.00 | 2.32 | unknown |
| *stpA* | 0.00 | 10.12 | negative regulation of transcription |
| *tatA* | 0.00 | 1.54 | protein transport by the Tat complex |
| *tatE* | 0.00 | 2.26 | protein transport by the Tat complex |
| *tolB* | 0.00 | 1.61 | bacteriocin transport |
| *trkA* | 0.00 | 1.46 | ion transport |
| *trxA* | 0.00 | 2.65 | cell redox homeostasis |
| *trxB* | 0.00 | 2.38 | cell redox homeostasis |
| *tyrP* | 0.00 | 3.14 | amino acid transmembrane transport |
| *ubiG* | 0.00 | 1.69 | hyperosmotic salinity response |
| *ureC_1* | 0.00 | 1.57 | unknown |
| *ureC_2* | 0.00 | 1.53 | unknown |
| *ureE_2* | 0.00 | 1.60 | unknown |
| *ureG_1* | 0.00 | 2.39 | unknown |
| *uvrD* | 0.00 | 2.67 | unknown |
| *wecG* | 0.00 | 1.51 | enterobacterial common antigen biosynthetic process |
| *wzy* | 0.00 | 1.54 | unknown |
| *wzzB* | 0.00 | 1.72 | lipopolysaccharide biosynthetic process |
| *xanP* | 0.00 | 3.45 | transmembrane transport |
| *yaaA* | 0.00 | 1.70 | response to hydroperoxide |
| *yajC* | 0.00 | 1.53 | protein insertion into membrane from inner side |
| *ybdG* | 0.00 | 1.71 | cellular response to osmotic stress |
| *ybhS* | 0.00 | 1.78 | drug transmembrane export |
| *ycdX* | 0.00 | 1.86 | bacterial-type flagellum-dependent swarming motility |
| *ychF* | 0.00 | 2.36 | response to oxidative stress |
| *yddG* | 0.00 | 3.12 | amino acid export |
| *ydgA* | 0.00 | 1.45 | bacterial-type flagellum-dependent swarming motility |
| *ydhL* | 0.00 | 2.40 | response to hydrogen peroxide |
| *ydiV* | 0.00 | 1.53 | negative regulation of bacterial-type flagellum-dependent cell motility |
| *ydjY* | 0.00 | 4.61 | unknown |
| *yeaR* | 0.00 | 4.18 | unknown |
| *yebB* | 0.00 | 3.25 | unknown |
| *yecC* | 0.00 | 2.09 | unknown |
| *yecD* | 0.00 | 2.09 | unknown |
| *yedD* | 0.00 | 2.48 | unknown |
| *yfcC* | 0.00 | 3.78 | transmembrane transport |
| *yfeH* | 0.00 | 1.79 | transmembrane transport |
| *yfeX* | 0.00 | 2.74 | cellular oxidant detoxification |
| *yhcC* | 0.00 | 3.69 | 4 iron, 4 sulfur cluster binding |
| *yhcN* | 0.00 | 2.71 | cellular response to acidic pH |

| | | | |
|---|---|---|---|
| *yiaF* | 0.00 | 1.55 | symbiosis, encompassing mutualism through parasitism |
| *yijE* | 0.00 | 2.20 | amino acid transmembrane transport |
| *yjbH* | 0.00 | 3.13 | extracellular polysaccharide biosynthetic process |
| *yjdP* | 0.00 | 3.52 | unknown |
| *yjiX* | 0.00 | 3.60 | cellular response to DNA damage stimulus |
| *ynjD* | 0.00 | 1.94 | transmembrane transport |
| *yoaB* | 0.00 | 1.92 | single-species biofilm formation |
| *ypdA* | 0.00 | 1.83 | cell wall organization |
| *ytfB* | 0.00 | 1.66 | FtsZ-dependent cytokinesis |
| *ytfE* | 0.00 | 1.71 | iron incorporation into metallo-sulfur cluster |
